# Supplementary material for: Development of monoclonal antibodies against E1 protein of Chikungunya virus
Source: PLoS Negl Trop Dis. 2025 Dec 4;19(12):e0013794. doi: 10.1371/journal.pntd.0013794 (PMC12700421; doi:10.1371/journal.pntd.0013794)

**S1 Fig. Immunofluorescence Assay (IFA) Detection of Anti-CHIKV-E1 Antibodies in Immunized Mouse Serum**

C6/36 cells (ATCC, CRL-1660) infected with Chikungunya virus (CHIKV, Asian-genotype) were washed with 1×PBS and spread onto chamber slides (Nunc, Inc.; Cat. No. 177402). Uninfected C6/36 cells served as a negative control. Cells were fixed with 10% formaldehyde, incubated with rCHIKV-E1-immunized BALB/c mouse serum (1:100 dilution) at room temperature for 1 hour, and washed six times with PBS containing 0.1% Tween-20 (PBS-T). Bound anti-CHIKV-E1 antibodies were detected using Alexa Fluor 488-conjugated goat anti-mouse IgG (H+L) secondary antibody (Thermo Fisher Scientific, Cat. No. A-11001) for 30 minutes at room temperature. After six PBS-T washes, cells were mounted with VECTASHIELD mounting medium containing 4’,6-diamidino-2-phenylindole (DAPI) (Vector Labs, Cat. No. H-1500-10) and imaged using an Olympus BX53 light microscope with a 20× objective. (A) rCHIKV-E1-immunized BALB/c mouse serum staining CHIKV-infected C6/36 cells, showing specific fluorescence; (B) rCHIKV-E1-immunized mouse serum staining uninfected C6/36 cells, showing no fluorescence.


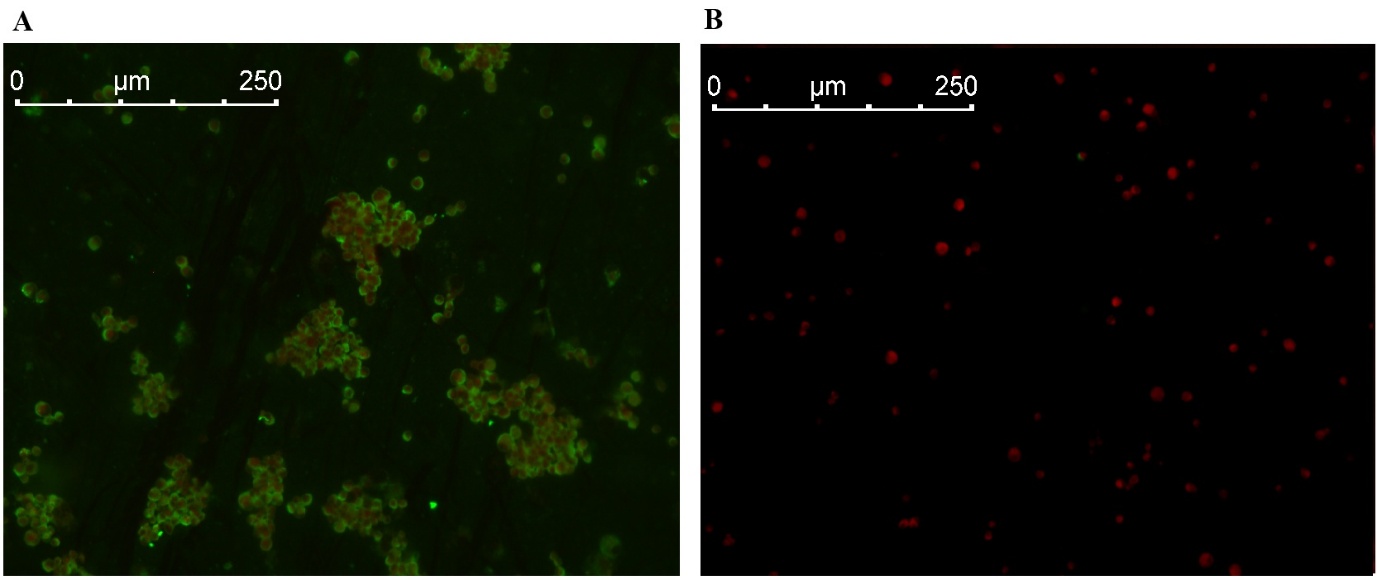

Supplement: S1 Fig — (DOCX) [file pntd.0013794.s005.docx]
