# Supplementary material for: Development of monoclonal antibodies against E1 protein of Chikungunya virus
Source: PLoS Negl Trop Dis. 2025 Dec 4;19(12):e0013794. doi: 10.1371/journal.pntd.0013794 (PMC12700421; doi:10.1371/journal.pntd.0013794)
Supplement: Attachment — Submitted filename: Response to Reviewers PNTD-D-25-00525.doc [file pntd.0013794.s007.doc]

**Point-to-Point Response**

**Manuscript number:** PNTD-D-25-00525

### ****Response to Reviewer #1****

1. The main weakness of this study is that the system was not evaluated using human serum samples. Without such validation, it is difficult to trust its suitability for diagnostic purposes.

**Response 1:** We completely concur with the reviewer's assertion regarding the inclusion of human serum samples in this study. We have gathered and examined 271 human serum samples (84 confirmed CHIKV-positive, 94 confirmed DENV1-positive, and 93 negative controls) to verify the accuracy of our ELISA method. The assay achieved a sensitivity of 94.05% (95% CI: 87.3–97.3) and a specificity of 98.4% (95% CI: 95.3–99.5). The results indicate that our test exhibits consistent reliability with human samples, showcasing elevated sensitivity and specificity. The additional data have been incorporated into the Results section, as well as Figures 4C and 4D, and Table 2.

2. The authors should test at least a small panel (e.g., 10 human serum samples) to verify the reliability of the ELISA system.

**Response 2: We would like to thank the reviewer for the comment.** We have incorporated 84 human serum samples. The amended manuscript's results and discussion section detail the findings, which validate the assay's diagnostic efficacy.

3. Since the same recombinant CHIKV E1 antigen was used for mouse immunization to generate monoclonal antibody clones, the ELISA system may be adequate for mouse-derived samples. However, the authors must demonstrate its applicability using human CHIKV-infected serum samples.

**Response 3:** We fully agree with the reviewer's point. We have now confirmed the assay utilizing human CHIKV-positive and negative serum samples. The findings endorse the utility of our ELISA for human diagnostic applications.

4. Among the monoclonal antibodies generated, the authors should clarify the criteria used to select the antibody for HRP conjugation.

**Response 4: We apologize for the problem. A section has been incorporated in the methods detailing the selection criteria: the antibody selected for HRP conjugation demonstrated the highest binding affinity in ELISA, robust reactivity in IFA, and no cross-reactivity with other arboviruses.**

5. In Table 1, some clones are positive for ELISA and IFA but negative for WB. The authors need to explain this discrepancy.

**Response 5:** Recombinant proteins utilized in immunoblotting are subjected to a temperature of 100°C for 10 minutes, thereafter separated by SDS-PAGE gel, and transferred to nitrocellulose membranes for interaction with the respective antibodies. This leads to linearization, potentially causing diminished reactivity and low affinity with certain antibodies that recognize conformational epitopes, hence yielding unfavorable results. ELISA and IFA utilize untreated recombinant proteins or native structural proteins, hence contrasting yet complementing one another.

6. The statement that there is no CHIKV outbreak in China is incorrect. In 2025, a CHIKV outbreak occurred in China. Please refer to the following sources:
<https://sangerinstitute.blog/2025/08/07/chikungunya-virus-in-china/>
<https://www.nature.com/articles/d41586-025-02794-2>

**Response 6:** We sincerely apologize for this oversight. We have corrected the statement in the Introduction and Discussion and cited the provided references.

7. As CHIKV outbreaks have also been reported in countries neighboring China, the authors should consider collaborating with regional partners to validate their ELISA system using human serum samples from outbreak settings.

**Response 7:** We fully concur with this proposal. The subtypes of Chikungunya virus (CHIKV) principally consist of the East/Central/South African (ECSA) genotype, the Asian genotype, the West African genotype, and the Indian Ocean genotype. The ECSA genotype is among the oldest and has evolved into the Indian Ocean genotype. The Asian genotype is prevalent in Asia and the Americas, whereas the West African genotype is predominantly located in Africa. The ECSA genotype is currently predominant in China. We are actively pursuing international collaboration and anticipate that our CHIKV antigen detection method will get broader validation and support.

### ****Response to Reviewer #2****

**Comment:**
From the pdf file that I received, the quality was far from excellent, please provide better ones.

**Response:** We regret the substandard quality of the figures in the initial submission. All figures have been processed using the PLOS NAAS tool and conform to the requisite criteria for resolution and format.

### ****Journal Requirements****

We confirm that all references suggested by the reviewers have been evaluated. Where relevant, we have included appropriate citations.
